# Genetic trends in the Zimbabwe’s national maize breeding program over two decades

**DOI:** 10.3389/fpls.2024.1391926

**Published:** 2024-06-05

**Authors:** Ronica Mukaro, Davison Chaingeni, Clay Sneller, Jill E. Cairns, Lennin Musundire, Boddupalli M. Prasanna, Busiso Olga Mavankeni, Biswanath Das, Mable Mulanya, Walter Chivasa, Xavier Mhike, Thokozile Ndhlela, Nakai Matongera, Prince Muchapondwa Matova, Dean Muungani, Charles Mutimaamba, Dagne Wegary, Mainassara Zaman-Allah, Cosmos Magorokosho, Victor Chingwara, Dumisani Kutywayo

**Affiliations:** ^1^ Crop Breeding Institute, Department of Research & Specialist Services, Harare, Zimbabwe; ^2^ Department of Horticulture and Crop Science, The Ohio State University College of Food, Agriculture and Environmental Science, Columbus, OH, United States; ^3^ Global Maize Program, International Maize and Wheat Improvement Center (CIMMYT), Harare, Zimbabwe; ^4^ Accelerated Breeding Initiative (ABI)-Transform, International Maize and Wheat Improvement Centre (CIMMYT), Nairobi, Kenya; ^5^ Global Maize Program, International Maize and Wheat Improvement Centre (CIMMYT), Nairobi, Kenya; ^6^ Integrated Breeding Platform (IBP), International Maize and Wheat Improvement Centre (CIMMYT), Nairobi, Kenya; ^7^ Scientific and Industrial Research and Development Center (SIRDC), Harare, Zimbabwe; ^8^ Mukushi Seeds (Pvt) Ltd, Harare, Zimbabwe; ^9^ Research for Development (R4D), International Institute of Tropical Agriculture (IITA), Ibadan, Nigeria; ^10^ Tocek Investments (Pvt) Ltd, Harare, Zimbabwe

**Keywords:** breeding efficiency, genetic gain, genetic trend, maize, Zimbabwe

## Abstract

Monitoring genetic gains within breeding programs is a critical component for continuous improvement. While several national breeding programs in Africa have assessed genetic gain using era studies, this study is the first to use two decades of historical data to estimate genetic trends within a national breeding program. The objective of this study was to assess genetic trends within the final two stages of Zimbabwe’s Department of Research & Specialist Services maize breeding pipeline between 2002 and 2021. Data from 107 intermediate and 162 advanced variety trials, comprising of 716 and 398 entries, respectively, was analyzed. Trials were conducted under optimal, managed drought stress, low nitrogen stress, low pH, random stress, and disease pressure (maize streak virus (MSV), grey leaf spot (GLS), and turcicum leaf blight under artificial inoculation. There were positive and significant genetic gains for grain yield across management conditions (28–35 kg ha^-1^ yr^-1^), under high-yield potential environments (17–61 kg ha^-1^ yr^-1^), and under low-yield potential environments (0–16 kg ha^-1^ yr^-1^). No significant changes were observed in plant and ear height over the study period. Stalk and root lodging, as well as susceptibility to MSV and GLS, significantly decreased over the study period. New breeding technologies need to be incorporated into the program to further increase the rate of genetic gain in the maize breeding programs and to effectively meet future needs.

## Introduction

**Figure 1:**
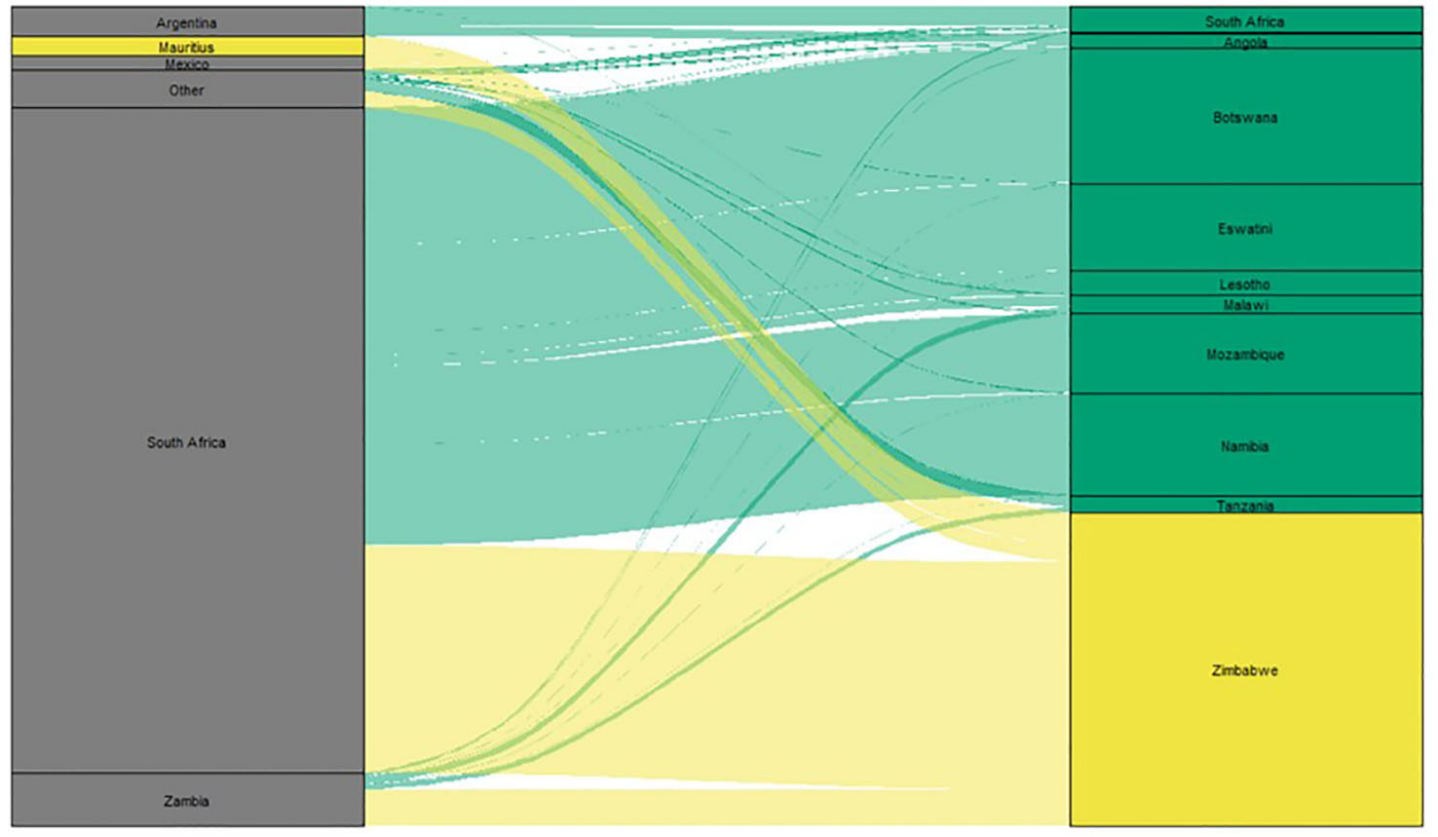
Alluvial diagram illustrating maize imports to southern African countries. Countries exporting maize to southern African countries are in the left column and southern African importing maize are in the right column. The height of the block is proportional to the volume of maize exported or imported by the corresponding country, and the width of the stream is proportional to the volume of maize traded between the two countries connected. Imports to Zimbabwe are highlighted in yellow, and all other imports are highlighted in green. (Data source: https://intracern.org, data is the average of 2020 and 2021).

Global food systems are under pressure from a growing population, changing climates, and conflicts (Chatzopoulos et al., 2021; Bentley et al., 2022). In southern Africa, maize is the primary cereal crop, accounting for up to three-quarters of the area under cereal production in 70% of the countries (FAO, 2024). In this region, the annual rainfall has been below average for half of the past decade, including a severe El Niño-induced drought, in 2016 (Frischen et al., 2020). The situation is likely to worsen with increasing climate variability (IPCC, 2022). Maize-based production systems in southern Africa were previously highlighted among the most vulnerable to climate change (Lobell et al., 2008; Tigchelaar et al., 2018). Without strong adaptation measures, climate change will reduce maize yields by over 15% in the region (Tesfaye et al., 2016; Zhai et al., 2021). Crises, such as the COVID-19 pandemic, have further exacerbated the vulnerability of food systems to external factors, and the need to build self-sufficiency (Tabe-Ojong et al., 2023). The majority of countries in Southern Africa do not produce enough maize to meet their demands annually and rely on imports. During favorable years, there is extensive regional maize trade and production deficits are primarily met through imports from neighboring countries (**Figure 1**). However, in drought-affected years, countries typically reduce or stop exports to meet their domestic requirements. In Zimbabwe, maize is produced on approximately 1 million hectares, accounting for over 70% of the area under cereal production (FAO, 2024). Nearly 80% of the grain yield variability in Zimbabwe over the past three decades has been related to climatic variability (Ray et al., 2015). Implementing strategies to significantly increase maize production and productivity in southern Africa, including Zimbabwe, is essential to build self-sufficiency and resilience against shocks.

Increasing maize yields and yield stability in a changing climate requires a range of interventions, including crop genetic improvement and improved agronomic management. Stress-tolerant maize has been identified as one of the most promising sustainable intensification strategies for maize in sub-Saharan Africa (SSA) (Jain et al., 2023). When compared to a range of technologies, the adoption of stress-tolerant maize generated the largest and most consistent positive impact on yield and economic outcomes (Jain et al., 2023). The ability of stress-tolerant maize hybrids to increase yields in farmers’ fields is dependent on both the rate of genetic gain for key traits and the rate of varietal replacement (Atlin et al., 2017). Zimbabwe has a long history of maize genetic improvement. In 1948, Zimbabwe was the first country in Africa to commercialize maize hybrids (Rusike and Donovan, 1995) and has commercialized the world’s first single-cross hybrids in 1960 (Derera and Musimwa, 2015; Musimwa and Derera, 2017). At present, Zimbabwe has the highest share of adoption of maize hybrids in SSA (Krishna et al., 2023). Hybrid maize seed development and deployment has been a major driver of production gains in maize in Zimbabwe (Rukuni, 2006). The Zimbabwe national maize breeding program was officially established in 1909, focusing on improving open-pollinated varieties (OPVs) (Weinmann, 1972; 1975). Successful OPVs included Hickory King (1900–1968), Salisbury White (1906–1969) and later Southern Cross (1945–1971) (Caulfield and Havazvidi, 1989). In 1932, the focus was shifted to the development of hybrid maize. The first maize hybrid registered by the program in 1948 was a top cross produced between an inbred N4 and an OPV (Mashingaidze, 2006), which became the first maize hybrid to be released in SSA (Rusike and Donovan, 1995). Three double-cross maize hybrids (SR11, SR13, and SR14) were subsequently released in 1949. The first single-cross maize hybrid (SR52) was released in 1960and became Zimbabwe’s most popular high-yielding variety (Derera and Musimwa, 2015; Musimwa and Derera, 2017). However, due to the high cost of producing single-crosses the breeding program shifted focus to three-way maize hybrids in 1971. This led to the release of two popular three-way hybrids, R201 and R215, in 1973 and 1974, respectively.

By the mid-1980s, maize yield gains associated with the use of the three-way maize hybrids (R201 and R215) were estimated to be around 46% in the large-scale and 30% in the small-scale farming sectors (Tattersfield, 1982; Mashingaidze, 2006). Due to continuous improvements in maize breeding, the Zimbabwe maize breeding program was rated as a success story in addressing the requirements of both large and small-scale farmers (Eicher, 1995). Maize breeding program progress was a key driver in inspiring an organized seed system, with the formation of the Southern Rhodesia Seed Maize Association in the 1940s (Weinmann, 1975; Havazvidi and Tattersfield, 2006). The association operated under a tripartite agreement with the government, farmers’ union, and seed association to deliver seeds of improved maize varieties to the farmers from 1970. The Zimbabwe seed industry continued to evolve after the liberalization of the sector in the 1980s, leading to the emergence of several private seed companies (Havazvidi and Tattersfield, 2006). Prior to liberalization, maize varietal improvement was primarily conducted by the government’s Department of Research and Specialist Services (DR&SS). With trade liberalization and the entrance of new players, the industry became competitive, and demand for products with better genetics increased. This also led to the private sector’s development of maize breeding programs. Over the past two decades, the DR&SS has released 17 hybrids (including white, yellow, quality protein maize, and provitamin A-enriched orange maize) and four OPVs, namely ZM309, ZM401, ZM421 and ZM521. In 2010, the hybrid ZS265 was released, and by 2021, it accounted for 12% of the seed market in Zimbabwe, based on certified seed production figures. In Zimbabwe, an estimated 44% of the total maize area is planted with new genetics (Chivasa et al., 2022), with an average of 7.67 varieties being released annually (Krishna et al., 2023) augmented by both public and private sector investment in breeding.

**Figure 2:**
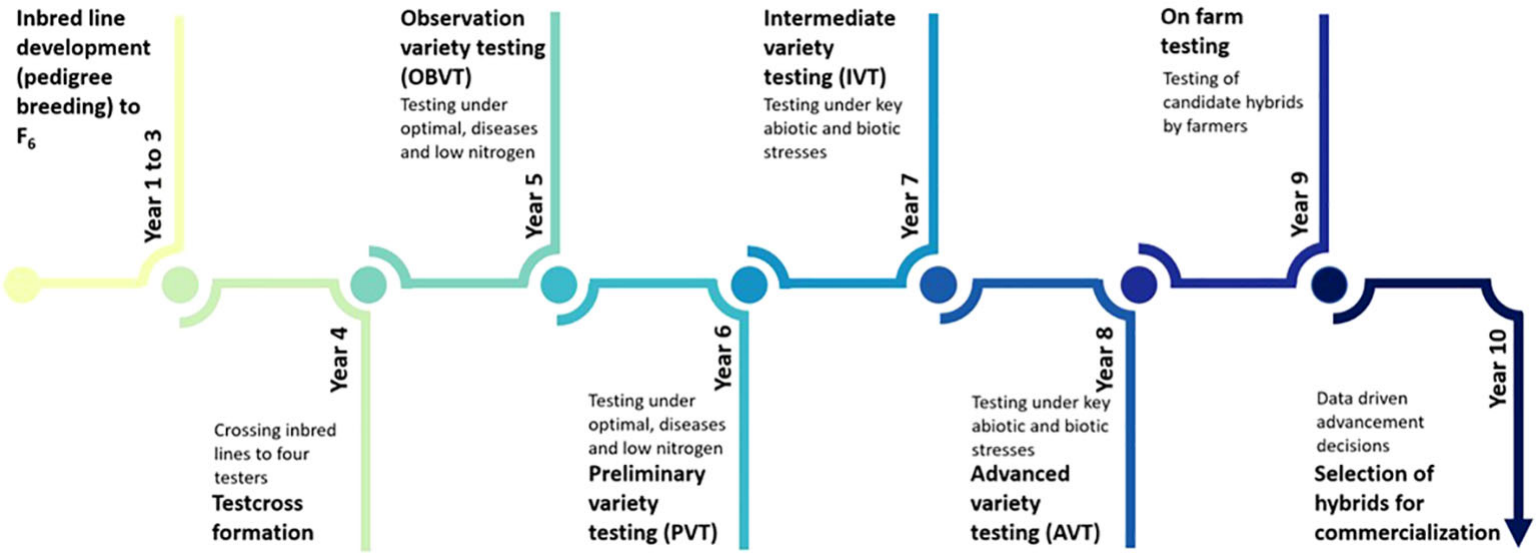
Overview of the Department of Research & Specialist Services maize breeding pipeline from inbred line development, testcross formation, on-station variety testing, on farm testing and finally the commercialization of promising hybrid. Candidate hybrids are advanced from observation variety trials (OVT) through to advanced trials (AVT) based on grain yield relative to commercial checks. The final stage of testing prior to commercialization in conducted by farmers within the target population of environments.

The DR&SS maize breeding program primarily targets drought-prone and low-input environments. The must-have traits include grain yield under drought, low nitrogen stress, tolerance to grey leaf spot (GLS, *Cercospora zea-maydis*), maize streak virus (MSV) and turcicum leaf blight (TLB), *Exserohilum turcicum*), reduced lodging and good husk cover. Tolerance to low pH soils was included as a must-have trait between 2010 and 2014. In the DR&SS maize breeding strategy, parents are selected, crossed, and progeny advanced to F_6_ (**Figure 2**). The development of inbred lines by selfing to F_6_ takes approximately 3.5 years, with two seasons per year. Inbred lines are test-crossed to four testers and candidate hybrids evaluated in observation trials (OBVT) few environments. The most promising candidate hybrids are advanced to preliminary variety trials (PVT), then intermediate variety trials (IVT) and finally to the advanced variety trials (AVT). Advancement of candidate hybrids to the next stage is primarily based on grain yield across locations and disease tolerance relative to commercial checks. The number of locations used increases as we advance from one stage to another and the number of candidate hybrids per stage decreases. In the IVT and AVT trials are conducted under optimal, managed drought stress, low nitrogen stress, random stress, artificially inoculated MSV, GLS and TLB disease pressure and low pH (less than pH 4 in Marondera and less than pH 5.6 in Bindura). The final stage of testing is conducted with smallholder farmers (on-farm evaluation) within the target population of environments. Candidate hybrids which grain yield more than the commercial checks, with farmer-preferred traits, are made available to seed companies for licensing and commercialization.

While private-sector crop breeding programs have historically focused on monitoring breeding progress (e.g. Duvick, 2005), public-sector breeding programs’ key performance indicators were primarily focused on varietal release (Atlin et al., 2017). Recently, there has been an increasing emphasis on monitoring genetic gains within public sector breeding programs focused on maize (Masuka et al., 2017a, Masuka et al., 2017b; Kebede et al., 2020; Prasanna et al., 2022; Menkir et al., 2022; Asea et al., 2023; Mazibuko et al., 2024), rice (Khanna et al., 2022; Rahman et al., 2023), wheat (Gerard et al., 2020; Mondal et al., 2020), and cassava (Manze et al., 2021). “Era studies”, where varieties of different ages (released in different years) are grown in a common trial across key environments, provide an important understanding and unbiased estimates of genetic gain, avoiding environmental and climate confounding effects. However, era studies represent a significant cost to breeding programs, while fewer genotypes are evaluated and there is less coverage of the target population of environments (Seck et al., 2023). For resource-constrained breeding programs, estimating genetic trends using historical data allows critical budgets to be utilized for population improvement and product advancement. Unfortunately, many breeding programs are unlikely to have access to extensive historical records due to high staff turnover, lack of digitized data, and stage-gate advancement across years.

While understanding current gains through selection is important to individual breeding programs, in the broader context, quantifying current gains within maize breeding programs in SSA is central to understanding if grain yield trends are sufficient to meet future demands in the region (Ray et al., 2013) and their ability to contribute to climate adaptation strategies (Challinor et al., 2016). The primary aim of this study was therefore to estimate genetic trends in Zimbabwe’s DR&SS maize breeding pipeline using historical data from 2002 to 2021. A secondary aim was to identify key steps to improve breeding efficiency within the pipeline.

## Materials and methods

### Product development and evaluation

Key founder inbred lines used extensively within the DR&SS maize breeding program for population development include 2Kba, 2N3d, K64r, M162W, NAW5885, N3–2.3.3, SV1P, RS61P and WCOBY1P (Ndhlela et al., 2015). The relative importance of lines changed over time as new improved lines were developed by DR&SS and sourced from national, regional, and international breeding programs. SV1P was recently identified as a key inbred line for tolerance for fall army worm (Matova et al., 2022). Recently donor lines with tolerance to heat stress have been incorporated into the program to enrich tolerance to heat stress within new populations formed (Mukaro et al., 2023). Heterotic groups included the broad CIMMYT A and B classification, Southern Cross (SC) and Northern Cross (NC). The maturity group targeted by the breeding program was early to intermediate. While commercial checks changed throughout the two decades of trials, commercial checks were present up to 18 years reflecting their popularity on the commercial markets (**Supplementary Table 1**).

Data from 2002 to 2021 IVTs and AVTs was used in this analysis. The number of entries per trial in the IVT ranged from 15 to 76, while the number of entries in the AVT ranged from 10 to 56 (**Table 1**). Commercial hybrids and OPVs from private seed companies and the International Maize and Wheat Improvement Centre (CIMMYT) were included as checks. A total of 716 varieties in the IVT and 398 varieties in the AVT were evaluated during the study period (**Table 1**).

**Table 1:** Summary of the years, tests and number of experiments and entries in the intermediate variety trials (IVT) and advanced variety trials (AVT) data sets of the Department of Research & Specialist Services (DR&SS) maize breeding program.

IVT				AVT			
Year	Test^Ϯ^	No. of experiments	No. of entries	Year	Test	No. of experiments	No. of entries
2002	IVT	5	40	2002	AVT	6	40
2003	IVT	7	40	2003	AVT	8	40
2004	IVT	6	40	2004	AVT	9	39
2005	IVT	6	40	2005	AVT	7	40
2006	IVT	8	40	2006	AVT	11	40
2007	IVT	7	40	2007	AVT	9	40
2008		0	0	2008		0	0
2009	IVT	1	32	2009	AVT	1	32
2010	IVT	8	32	2010	AVT	5	56
2011	IVT	11	42	2011	AVT	7	42
2012	EM	6	25	2012	AVT	12	40
2012	ML	5	15	2013	AVT	10	40
2013	EDT	4	64	2014	EDT	9	54
2013	MDR	5	80	2014	LDT	7	40
2014	EDT	5	40	2014	SB	2	24
2014	LDT	1	76	2015	EDT	5	44
2015	EDT	3	25	2015	LDT	4	41
2015	LDT	5	30	2016	LDT	1	24
2016	DT	6	50	2017	EMDT	5	39
2017		0	0	2017	LDT	4	10
2018	IVT	3	60	2017	MLIR	4	15
2019	IVT	2	35	2018	EMDT	6	18
2020	IVT	2	76	2018	LDT	7	10
2021	DTML	1	42	2018	MLIR	5	15
				2019	EMDT	3	12
				2019	EMH	6	19
				2019	MLIR	3	25
				2020	ELTA	3	36
				2021	ELMH	3	24

IVT, Intermediate variety trial; AVT, Advanced variety trial; DT, Drought tolerant; DTM, EDT, Early drought tolerant; EM, EM, EMDT, Early to medium drought tolerant; LDT, Late drought tolerant; MDR, multiple disease resistance; ML, MLIR, SB, Stemborer.

### Field trials

In this study, a total of 107 trials were used from the IVT, and 162 from the AVT (**Table 1**). In the IVT the number of trials per year ranged from one (2009 and 2021) to 11 (2011 and 2012), with no data in 2008 and 2017 (**Table 1**). In the AVT the number of trials per year ranged from one (2006 and 2009) to 18 (2018); there was no AVT data in 2008 (**Table 1**). In all experiments an alpha lattice design was used, replicated three times. Trials were planted in two-row plots, with 0.75 m between rows and 0.25 m between plants, with a final plant density of approximately 53 300 plants ha^-1^. Row length varied across locations, and plot size ranged from 3.2 to 6.38 m^2^.

**Figure 3:**
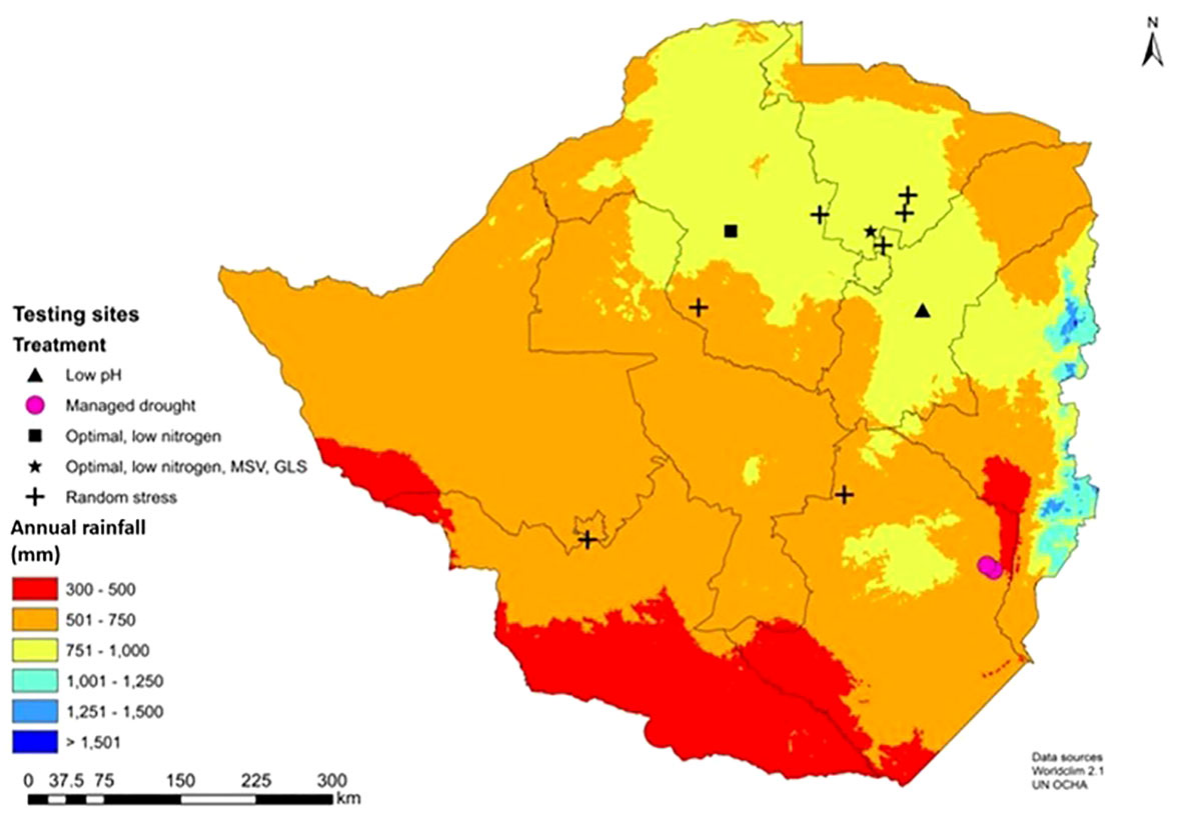
Key phenotyping sites for abiotic (managed drought, random stress and low nitrogen), biotic (maize streak virus, MSV; grey leaf spot, GLS) and optional within the Department of Research and Specialist Services (DR&SS) maize breeding program in Zimbabwe.

Experiments were conducted across 12 locations in Zimbabwe (**Figure 3**). Optimal experiments were planted in the main season in high-rainfall locations. Nationally recommended fertilizer rates were applied and recommended weed and insect control followed. If required, supplemental irrigation was applied to ensure trials did not encounter drought stress at any point. Managed drought stress experiments were planted in the winter (dry) season and recommended fertilizer rates, weed and insect control followed. Trials were fully irrigated approximately two weeks before flowering to induce drought stress at anthesis-stage, the most susceptible stage to drought (Bänziger et al., 2000). Irrigation was withheld until two weeks after flowering. Random stress experiments were planted in the main (wet) season in drought-prone regions (natural regions IV and V) without supplemental irrigation. In general, experiments experienced several periods of drought throughout the growing season, which varied each year depending on the nature of the season (Bänziger et al., 2006). Low nitrogen experiments were established in fields depleted of native and accumulated nitrogen by continuously planting maize and removing all biomass at harvest, without adding inorganic or organic nitrogen fertilizer for more than 10 cropping seasons (Zaman-Allah et al., 2015). The aim was to deplete fields of nitrogen to reduce yields by approximately 60% relative to optimal conditions (Das et al., 2018).

MSV, TLB and GLS are endemic diseases throughout Zimbabwe and the market requires increasing levels of tolerance to these diseases. Disease screening was primarily conducted under natural infestation in disease hot spots Commercial checks, which were highly susceptible to these diseases, were included in IVT and AVT experiments to provide a benchmark for selection of candidate hybrids to advance to the next stage. In 2012, capacity for artificial inoculation of MSV, TLB and GLS were established in Harare to ensure more uniform disease pressure. By 2017, two-thirds of the experiments for MSV, TLB and GLS were under artificial inoculation (with one third still conducted under natural infestation in disease hotspots). Experiments artificially inoculated for MSV, GLS and TLB accounted for less than 30% of the total number of disease trials in this study. Artificial inoculation was conducted at Harare research station to ensure uniform disease pressure. Artificial inoculation for MSV and TLB followed the protocol described by Karavina et al., 2014. For MSV, viruliferous *Cicadulina mbila* (Naude) leafhoppers were raised and released onto the leaf whorl of each individual plant. Three hoppers were placed per plant to facilitate the spread of MSV. For TLB, all plants were inoculated with a spore suspension of *Helminthosporium turcicum*. The suspension was sprayed onto the leaf whorl of all plants three weeks after sowing. To facilitate sport germination and disease infection, a light irrigation was applied after inoculation. For artificial inoculation of GLS, a spore suspension of *Cercospora zea-maydis* was produced using infested leaves harvested the previous season. The spore suspension was sprayed into the leaf whorl of every plant approximately two weeks after crop emergence. To enhance disease infection, border rows of a highly susceptible hybrid were planted around the trial (Sibiya et al., 2012).

### Measurements

For MSV, GLS and TLB, disease severity was scored on a plot basis using a visual scale of 1 to 5 where 1 represents no disease or traces of disease, 2 = lesions present on lower leaves but little or no disease above the ear leaf, 3 = disease present on most leaves with some lower leaves dead, 4 = lower leaves dead and numerous lesions on all upper leaves, and 5 = nearly all leaf tissue killed. Days to anthesis and silking were recorded when 50% of the plants had shed pollen, and 50% of the plants had silks, respectively. Before harvesting, plant height and ear height were measured on two representative plants per plot. Ear position was calculated by dividing ear height by plant height. At harvest, the number of ears and plants per plot were counted to calculate ears per plant. The number of plants per plot with root and stalk lodging were counted and expressed as a percentage of the total number of plants in a plot. Grain texture was visually estimated per plot using a 1 to 5 scale, where 1 represents flint, round crown kernels, and 5 represents dent kernels with a floury endosperm. Ear rots were visually estimated per plot using a visual scale of 1–5, where 1 represents no ear rots, and 5 represents very heavy infection. At harvest, two plants from either end were eliminated, and the rest of the plants were hand-harvested, and grain yield and moisture content were measured. Grain yields were adjusted to 12.5% moisture content and expressed in kg ha^-1^. Not all traits were assessed in each experiment, with a larger number of traits assessed in the AVT than in the IVT.

### Statistical analysis

All of the analyses were done separately for the IVT and the AVT. An analysis of variance was performed within each experiment using the model (Equation 1):


(1) $$y_{ij}=\mu +g_{i}+r_{j}+e_{ij}$$

where $y_{ij}$ is the mean phenotypic value of the trait within an experiment, $g_{i}$ is the random effect of the *i* ^th^ genotype with $g_{i}\sim N(0,\;\sigma_{g_{i}}^{2})$ , $r_{j}$ is the random effect of the *j* ^th^ replication with $r_{j}\sim N\left(0,\;\sigma_{r_{j}}^{2}\right)$ , and $e_{ij}$ is the residual error with $e_{ij}\sim N\left(0,\;\sigma_{e_{il}}^{2}\right)$ .

Analysis of variance was conducted over all experiments within each year using the model (Equation 2):


(2) $$y_{ijk}=\mu +g_{i}+x_{k}+r{\left(x\right)}_{jk}+gx_{ik}+\;e_{ijk}$$

where $y_{ijk}$ is the mean phenotypic value of the trait within an experiment within a year, $x_{k}$ is the random effect of the *k* ^th^ experiment with $x_{k}\sim N(0,\;\sigma_{x}^{2})$ , $r{\left(x\right)}_{jk}$ is the random effect of the *j* ^th^ replication nested in the k^th^ experiment with, $r{\left(x\right)}_{jk}\sim N\left(0,\;\sigma_{r\left(x\right)}^{2}\right)$ , and $e_{ijk}$ is the residual error with $e_{ijk}\sim N\left(0,\;\sigma_{e}^{2}\right)$ .

Entry mean heritability was calculated within each experiment as (Equation 3):


(3) $$H=\frac{\sigma_{g}^{2}}{\sigma_{g}^{2}+(\frac{\sigma_{e}^{2}}{r})}$$

where, $\sigma_{g}^{2}$ , $\sigma_{e}^{2}$ are the genetic and error variances, and r is the number of replications. Entry mean heritability within a year was calculated as (Equation 4):


(4) $$H=\frac{\sigma_{g}^{2}}{\sigma_{g}^{2}+\langle \frac{\sigma_{gx}^{2}}{x}\rangle +\langle \frac{\sigma_{e}^{2}}{xr}\rangle }$$

where $\sigma_{gx}^{2}$ is the genotype by experiment variance and x is the number of experiments in the trial.

The standardized residual for each plot within each experiment was calculated as e_ij_/σ_e_ where e_ij_ is the residual for the i^th^ genotype, and j^th^ replication and σ_e_ is the error standard deviation. Plots where the absolute value of e_ij_/σ_e_ was greater than 1.75 were removed from analyses. For every trait, data from experiments with H< 0.20 was excluded from further analysis. A mixed model analysis was then conducted to obtain the Best Linear Unbiased Estimates (BLUE) of each entry using the model (Equation 5):


(5) $$y_{ijklm}=\mu +g_{i}+\;c_{j}+t_{k}+\;x{\left(t\right)}_{kl}+r{\left(x\right)}_{lm}+\;\;gt_{ik}+\;gx{\left(t\right)}_{ikl}+\;\varepsilon_{ijkl}$$

where $y_{ijklm}$ is the phenotypic value for genotype *i* tested in control group *j*, xperiment *k*, experiment l, and replication *m*. μ is the overall mean; $g_{i}$ is the fixed effect of the i^th^ genotype, $c_{j}$ is the fixed effect of the *j^th^* control where *j*=1 for the control population consisting of the checks and *j*=2 for the lines group; $t_{k}$ is a random effect for the *k^th^* experiment with $t_{k}\sim N(0,\;\sigma_{t_{k}}^{2})$ ; $x{\left(t\right)}_{kl}$ is a random effect for the l*^th^* experiment nested in the k^th^ experiment with $x{\left(t\right)}_{kl}\sim N(0,\;\sigma_{x{\left(t\right)}_{kl}}^{2}$ ); *gt_ik_* is the random effect of the interaction of the i^th^ genotype and k^th^ experiment with $gt_{ik\;}\sim N(0,\;\sigma_{gt_{ik}}^{2})$ ; *gx(t)_ikl_* is the random effect of the interaction of the i^th^ genotype and l^th^ experiment nested in the k^th^ experiment with $gx{\left(t\right)}_{ikl\;}\sim N(0,\;\sigma_{gt_{ikl}}^{2})$ ; $\;r{\left(x\right)}_{lm}$ is a random effect of the m^th^ replication nested in the l^th^ experiment with $r{\left(x\right)}_{lm}\sim N(0,\;\sigma_{r{\left(x\right)}_{lm}}^{2})$ ; and $\;\varepsilon_{ijklm.}$ Is the residual error with $\varepsilon_{ijklm}\sim N\left(0,\;\sigma_{\epsilon_{ijklm}}^{2}\right)$ . This analysis used data from all entries in the IVT or AVT, including non-candidate hybrids.

Genetic trends within the DR&SS breeding pipeline were estimated by regressing the BLUEs of DR&SS varieties only onto their first year of testing (FYT). The regression slope provides an estimate of genetic gain per the first year of testing in units of the trait. This slope was also expressed as a percentage of the grand mean of all candidate hybrids and checks. Data from experiments with heritability less than 0.20 was excluded from the analysis. Genetic trends were estimated across all experiments, within high yielding environments (experiment mean grain yield > 3000 kg ha^-1^), and within low-yielding environments (experiment mean grain yield< 3000 kg ha^-1^).

We estimated the relative efficiency of improving grain yield in low-yielding environments by selecting for grain yield in high-yielding environments. The BLUEs for grain yield were obtained using Equation 6 with data from low and high yielding environments. The phenotypic correlation of grain yield in the low-yielding and high-yielding environments (r_p_) was calculated. The genetic correlation (r_g_) was also calculated as:


(6) $$r_{g}=\;\frac{r_{p}}{\sqrt{h_{lgy}^{2}h_{hgy}^{2}}}$$

where $h_{lgy}^{2}$ and $h_{hgy}^{2}$ are heritability of grain yield in low-yielding and high-yielding environments, respectively. We used H as a surrogate for *h^2^* in Equations 6 and 7. The relative efficiency of indirect selection was then calculated as:


(7) $$RE_{lgy,hgy}=\;r_{g}\frac{h_{hgy}}{h_{lgy}}$$

The performance of the IVT candidate varieties advanced to the next year’s AVT were tracked. In the IVT and AVT sets, the performance of candidate varieties were assessed as 1) overall IVT experiments (IA) and all AVT experiments (AA), 2) only high-yielding IVT experiments (IH) and AVT experiments (AH), and 3) only low-yielding IVT experiments (IL), and only low yielding AVT experiments (AL). Correlations of performance in IA, IH, and IL with AA, AH, and AL for each pair of consecutive years with at least ten candidate varieties in common and with high-GY and low-GY environments were calculated. The top 20% of varieties in the IVT-based IA, IH, and IL, and compared their grain yield in AA, AH and AL experiments were selected. A t-test was conducted to see if there were significant differences between the means and the correlations.

## Results

### Trait means and repeatability

In this study, the percentage of check entries used was approximately 15% in the IVT and 36% in the AVT with a total of 107 IVTs and 162 AVTs. Average grain yield ranged from 200 kg ha^-1^ to 11,452 kg ha^-1^ in the IVT and from 103 kg ha^-1^ to 14,697 kg ha^-1^ in the AVT (**Table 2**). Forty IVT experiments and 72 AVT experiments had an average grain yield of less than 3,000 kg ha^-1^. Mean plant height across all IVT experiments was 202.9 cm compared to 189.1 cm across all AVT experiments. Similarly, the average ear height across all IVT was higher than the average ear height across the AVT. The average severity of MSV across all AVT experiments ranged from 1.0 to 3.9. For GLS, average disease severity ranged from 1 to 4.4 across all AVT experiments. The average severity of TLB across all AVT experiments ranged from 1.0 to 3.9 (**Table 2**).

**Table 2:** Summary across individual experiments of maize trait means, and heritability estimated within the intermediate variety trials (IVT) and advanced variety trials (AVT).

Set	Trait	# experiments	Trait means			Heritability			
			Mean	Minimum	Maximum	% experiments H<0.02	Mean	Minimum	Maximum
IVT	Grain yield (kg ha^-1^)	100	3994	200	11452	18.0	0.495	0.000	0.902
AVT	Grain yield (kg ha^-1^)	157	4013	103	14697	10.2	0.519	0.000	0.910
IVT	Moisture content (%)	90	14.3	7.3	25.8	30.3	0.375	0.000	0.921
AVT	Moisture content (%)	153	14.0	7.2	27.1	14.4	0.362	0.000	0.893
IVT	Plant height (cm)	96	202.9	79.6	308.8	23.7	0.506	0.000	0.904
AVT	Plant height (cm)	141	189.1	102.9	305.2	15.6	0.444	0.000	0.931
IVT	Ear height (cm)	92	98.6	31.2	158.8	23.4	0.509	0.000	0.932
AVT	Ear height (cm)	141	89.8	36.3	153.8	15.6	0.507	0.000	0.917
AVT	Ears per plant	87	0.8	0.4	1.2	33.3	0.327	0.001	0.823
AVT	Ear position	64	0.5	0.3	1.1	10.9	0.469	0.028	0.902
AVT	Texture	68	2.6	1.2	5.0	27.9	0.430	0.000	1.000
AVT	Stalk lodging	131	0.6	0.0	3.5	58.0	0.194	0.000	0.819
AVT	Root lodging	129	1.7	0.0	19.1	53.5	0.228	0.000	0.757
IVT	Days to silking	102	73.3	52.9	190.2	9.9	0.671	0.000	0.981
AVT	Days to silking	127	71.2	53.7	104.6	7.9	0.690	0.000	0.988
IVT	Days to anthesis	100	68.2	51.6	133.4	11.8	0.705	0.000	0.982
AVT	Days to anthesis	142	69.1	51.2	148.9	9.9	0.683	0.000	0.983
AVT	Maize streak virus (MSV)	21	2.0	1.0	3.9	23.8	0.361	0.001	0.727
AVT	Grey leaf spot (GLS)	31	1.7	1.0	4.4	51.6	0.244	0.001	0.803
AVT	Turcicum leaf blight (TLB)	34	1.9	1.0	3.9	38.2	0.272	0.001	0.737
AVT	Ear rot	74	2.3	1.0	5.0	36.5	0.338	0.000	0.900

The most heritable traits were grain yield, plant height, ear height, ears per plant, grain texture, silking date and anthesis date. These traits had an average experiment heritability greater than 0.44 each, and less than 27.9% of the experiments had a heritability<0.2. In contrast, the disease-related traits (GLS, TLB, MSV, and ear rot) and root and stalk lodging had an average H<0.37 and had at least 33% of the experiments with H<0.20 (**Table 2**).

**Figure 4:**
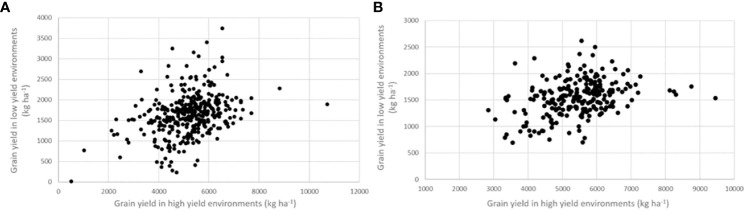
Relationship between grain yield in high environments (mean yield < 3000 kg ha^-1^) and low yield environment (average yield > 3000 kg ha^-1^) in the **(A)** intermediate variety trials (IVT) and **(B)** advanced variety trials (AVT).

The relative efficiency (RE) of improving grain yield in low yield experiments by selecting for grain yield in high-yield experiments is presented in **Table 3**. Phenotypic correlations between the low- and high-yielding environments were 0.348 in the IVT and 0.356 in the AVT (**Figure 4**), while genetic correlations were 0.557 and 0.634 in the IVT and AVT, respectively. For grain yield, heritability, assuming four experiments and three replications, was 35% greater in the high-yielding experiments of the IVT and 66% greater in the high-yielding experiments of the AVT than in the corresponding low-yielding environments. These differences in average heritability occurred despite eliminating experiments with H< 0.20 where such experiments were more frequent in low- versus high-yield experiments. These estimates produced a relative efficiency of improving grain yield in the low-yielding environments by selecting for grain yield in high-yielding environments of 0.753 in the IVT and 1.05 in the AVT.

**Table 3:** Summary of genetic trends (as expressed as slope) results from regressing the BLUEs of each candidate hybrid onto its first year of testing.

Trial	Trait	# Entries	INT	Slope	Prob	R^2^	Mean	Slope a % Mean
IVT	Grain yield, all (kg ha^-1^)	558	-55936	28.09	0.001	0.0222	3557	0.79
AVT	Grain yield, all (kg ha^-1^)	251	-70561	35.02	<0.001	0.0982	3954	0.89
IVT	Grain yield, high yield (kg ha^-1^)	550	-33459	17.02	0.048	0.0582	4519	0.38
AVT	Grain yield, high yield (kg ha^-1^)	246	-123697	61.38	<0.0001	0.1226	5651	1.09
IVT	Grain yield, low yield (kg ha^-1^)	182	237.8	-0.085	0.988	0.000	1591	-0.01
AVT	Grain yield, low yield (kg ha^-1^)	242	-31628	15.89	<0.0001	0.065	1283	1.24
IVT	Moisture content (%)	512	-123	0.061	<0.001	0.118	13.3	0.46
AVT	Moisture content (%)	256	-105	0.050	<0.001	0.089	13.9	0.37
IVT	Plant height (cm)	549	815	-0.401	<0.001	0.003	196	-0.20
AVT	Plant height (cm)	256	209	-0.100	0.360	0.003	201	-0.05
IVT	Ear height (cm)	555	-104	0.048	0.546	0.001	106	0.05
AVT	Ear height (cm)	256	-10.3	0.002	0.981	0.000	94	0.00
AVT	Ear position	179	0.27	0	0.837	0.000	0.53	0.00
AVT	Ears per plant	183	-26	0.013	<0.001	0.252	0.78	1.67
AVT	Texture	182	87.4	-0.043	<0.001	0.094	2.51	-1.71
AVT	Stalk lodging	209	32	-0.016	0.034	0.022	0.81	-1.98
AVT	Root lodging	237	223.5	-0.111	<0.001	0.225	2.71	-4.10
IVT	Days to silking	576	151	-0.075	0.116	0.004	69.7	-0.11
AVT	Days to silking	236	-186	0.093	0.006	0.032	70.6	0.13
IVT	Days to anthesis	558	468.3	-0.232	<0.0001	0.084	5.16	-4.50
AVT	Days to anthesis	256	-251	0.125	<0.001	0.068	67.8	0.18
AVT	Maize streak virus (MSV)	122	56.3	-0.028	0.032	0.038	2.10	-1.33
AVT	Grey leaf spot (GLS)	170	22.6	-0.011	0.048	0.023	2.37	-0.46
AVT	Turcicum leaf blight (TLB)	191	22.7	0.012	0.014	0.032	2.04	0.59
AVT	Ear rots	170	106.3	-0.053	<0.001	0.096	1.83	-2.90

Analyses were performed using data from the intermediate variety trials (IVT) and advanced variety trials (AVT). The grand mean of all the Department of Research and Specialist Services (DR&SS) entries is also presented.

On average, there were 19 experimental hybrids tested in the IVT and AVT in consecutive years, with a range of 11 to 42 hybrids tested in successive years. The average correlation of grain yield from all IVT experiments (IA), high-yielding IVT environments (IH) or low-yielding IVT environments (IL) with grain yield in all AVT experiments (AA), high-yielding AVT environments (AH) or low-yielding AVT environments (AL) were calculated (**Table 4**). There was no significant difference (p>0.05) in the correlation of grain yield in the IA, IH, or IL experiments with grain yield in the AA, AH, or AL experiments: all three types of IVT experiments were equally predictive of performance in the AA, AL, or AH experiments. Numerically, IA and IH experiments were better predictors of AVT performance than IL experiments. Correlations of IVT performance were the lowest when trying to predict AL. There was no significant difference (p>0.05) among the means of the selections from IA, IH, or IL experiments in the AA, AH, or AL experiments: selections for all three types of IVT experiments were statistically the same in AA, AL, or AH experiments (**Table 4**). Numerically, the selection from IA and IH experiments was better than from IL experiments.

**Table 4:** Estimates of variance components (V_g_, genotypic variation; V_ge_, genotype x environment variation; V_error_, residual variance), heritability (H), the phenotypic and genetic correlation of grain yield in low and high yield experiments and the relative efficiency of improving grain yield in low yield experiments by selecting for grain yield in high-yield experiments.

	Intermediate variety trial	Advanced variety trial
Pearson correlation	0.348	0.356
V_g_ average low yield	122,251	50,438
V_ge_ average low yield	24,880	76,047
V_error_ average low yield	435,507	552,793
H - low yield	0.537	0.436
V_g_ average high yield	850,095	644,992
V_ge_ average high yield	207,967	558,872
V_error_ average high yield	1,565,672	1,093,746
H - high yield	0.725	0.725
Genetic correlation	0.557	0.634
Relative efficiency	0.753	1.055

### Genetic trends

**Figure 5:**
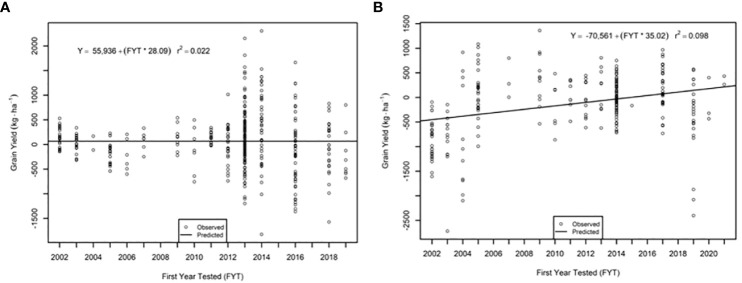
Genetic trend for grain yield across all environments in the **(A)** intermediate variety trial (IVT) and **(B)** advanced variety trial (AVT). Values presented are relative to the first year of testing of a specific entry.

**Figure 6:**
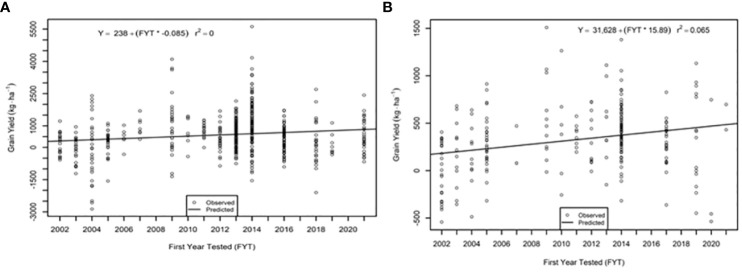
Genetic trend for grain yield under **(A)** low yield (< 3000 kg ha^-1^) environments and **(B)** high yield (> 3000 kg ha^-1^) environments in the intermediate variety trial (IVT). Values presented are relative to the first year of testing of a specific entry.

**Figure 7:**
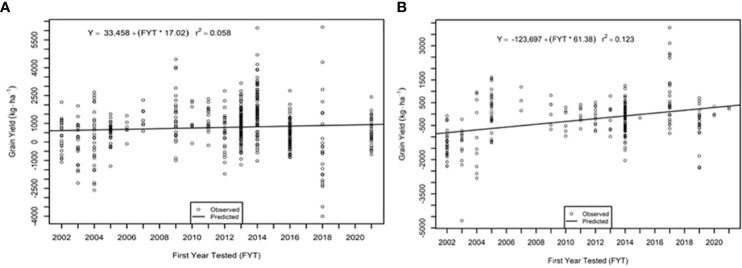
Genetic trend for grain yield **(A)** low yield (<3000 kg ha^-1^) environment and **(B)** high yield (> 3000 kg ha^-1^ environments in the advanced variety trial (AVT). Values presented are relative to the first year od testing of a specific entry.

The genetic trend for grain yield was greater in the AVT than in the IVT in all analyses. Across all management conditions grain yield increased significantly (p<0.05) by 28.09 kg ha^-1^ yr^-1^ in the IVT and by 35.02 kg ha^-1^ yr^-1^ in the AVT (**Figure 5**, **Table 5**). The genetic grain for yield in high-yielding environments increased significantly in the IVT at 17.02 kg ha^-1^ yr^-1^, with no significant trend in low-yielding environments (**Figure 6**). In the AVT grain yield increased significantly in the low-yielding environments by 15.89 kg ha^-1^ yr^-1^ and by 61.83 kg ha^-1^ yr^-1^ in high-yielding environments (**Figure 7**). The genetic increase was 3.86 times greater in the high-yielding AVT than in the low-yield experiments. The R^2^ values were low for all grain yield regressions, with the highest R^2^ of 0.123 in the high-yielding AVT.

**Table 5:** Summary of the performance of intermediate variety trial (IVT) candidate hybrids that are advanced to the advanced variety trial (AVT) trial.

Source of IVT data or selections	Average grain yield in the AVT of top 20% IVT selection (kg ha^-1^)			Correlation of grain yield between the IVT and AVT sets		
	All AVT Exp. (AA)	Low yield AVT Exp. (AL)	High yield AVT Exp. (AH)	All AVT Exp. (AA)	Low yield AVT Exp. (AL)	High yield AVT Exp. (AH)
All IVT Exp. (IA)	3932	1746	6356	0.434	0.335	0.329
Low-yield IVT Exp. (IL)	3663	1592	6140	0.212	0.271	0.122
High-yield IVT Exp. (IH)	3954	1711	6426	0.394	0.272	0.311

Moisture content increased significantly by 0.05 and 0.06% per year in the IVT and AVT, respectively. The regression from the IVT is one of just two analyses to produce an R^2^ greater than 0.2 (**Table 3**). Plant height significantly decreased in the IVT by 0.4 cm yr^-1^. There was no significant change in plant height in the AVT. There was no significant change in ear height in both the IVT and AVT over the study period. Ears per plant significantly increased in the AVT by 0.013 yr^-1^ (**Table 5**). Root and stalk lodging significantly declined over the study period in the AVT by -0.111 yr^-1^ and -0.016 yr^-1^, respectively. There was no significant change in silking date within the IVT, however, within the AVT silking date decreased significantly by 0.093 days yr^-1^. Anthesis date decreased by 0.232 days yr^-1^ in the IVT and increased by 0.125 days yr^-1^ in AVT. There was no significant change in resistance to GLS and TLB during the study period. However, MSV and ear rots scores decreased by -0.028 and -0.053 yr^-1,^respectively.

## Discussion

Without measures, increasing climate variability is highly likely to significantly reduce maize yields in SSA, particularly in Southern Africa (Lobell et al., 2008; Tesfaye et al., 2016; Muthuvel et al., 2023). Access to a steady stream of incrementally improved stress tolerant maize varieties has been shown to be an important adaptation mechanism (Hansen et al., 2019; Jain et al., 2023). Unlike other regions of the world, national and international maize breeding programs in SSA are a primary source of germplasm for commercial hybrid maize seed companies (Langyintuo et al., 2010; Chivasa et al., 2022). This study is the first in Africa to quantify genetic trends in maize breeding using historical data from two decades. Asea et al. (2023) estimated genetic trends for grain yield and key agronomic traits in pre-commercial and commercial maize varieties in Uganda using data from 2008 to 2020. In the present study, genetic gains in grain yield were 28.1 kg ha^-1^ yr^-1^ in the IVT and 35.0 kg ha^-1^ yr^-1^ in the AVT. The gain in grain yield was 28.1 kg ha^-1^ yr^-1^ in the IVT selected from the OBS and PVT. For varieties in the AVT the genetic gain was 35.0 kg ha^-1^ yr^-1^. Estimated genetic gain for grain yield were higher in the AVT than the IVT. While heritability of experiments was similar between the two stages, gains in the IVT and AVT result from the selection of candidate hybrids in the previous stage. Candidate hybrids in AVT are selected based on performance in IVT. The IVT candidate hybrids are selected from the observation and preliminary trials that are conducted at fewer locations per year than the IVT. Thus, the estimated genetic value of candidate varieties entering the IVT are likely to be less precise than the estimate of the genetic value of the experimental varieties entering the AVT, thus leading to lower genetic gain in the IVT than in the AVT.

When expressed as a percentage, gains in both low-yielding and high-yielding environments were similar to those reported in an era study for maize grain yield under drought stress (0.85% yr^-1^), random drought stress (0.85% yr^-1^) and low nitrogen stress (0.62% yr^-1^) in eastern and southern Africa (Masuka et al., 2017a). Genetic trends in maize grain yield in DR&SS were lower than Uganda’s National Agricultural Research Organization (NARO) maize breeding program, which reported that grain yield increased by 1.3% yr^-1^ over a twelve-year period using an era study (Asea et al., 2023). When expressed as a percent, genetic trends in grain yield were similar to the gains reported by Prasanna et al. (2022) under drought and low nitrogen stress, except for one breeding pipeline in Eastern Africa and one in Southern Africa. In absolute terms, genetic gains in grain yield were low, likely due to the focus on low-yielding environments that are similar to yields within the target population of environments (TPE). While average maize yields of smallholder farmers in Zimbabwe are increasing, they remain below 2 t ha^-1^ (ZimVAC, 2022). Over 43% of experiments had a grain yield of<3,000 kg ha^-1^ to ensure the selection for stress tolerance. While other breeding programs focusing on the same TPE also strongly emphasize selection for drought and low nitrogen stress tolerance, only half of the experiments are under abiotic stress (Prasanna et al., 2022). Furthermore, in Prasanna et al. (2022), less than 20% of experiments had a mean yield of less than 3,000 kg ha^-1^. Thus, the DR&SS maize breeding program occupies a unique space within maize breeding in Zimbabwe with its emphasis on stress-prone environments.

Genetic trends for grain yield were considerably lower in the low-yielding environments than in the high-yielding environments, in agreement with similar studies conducted in maize in the region (Masuka et al., 2017a, b; Prasanna et al., 2022; Asea et al., 2023; Tarekegne et al., 2023). Grain yield under stress is a key trait within the DR&SS product profiles, and it is an essential trait to increase yields in smallholder farmers’ fields. Lower gains in low-yielding environments were likely due, in part, to the lower heritability observed in low- versus high-yielding environments. Apart from low-N screening, abiotic stress phenotyping locations are at a significant distance from Harare, and thus experiment management is logistically more complicated and expensive. Therefore, testing in low yields sites should be done in AVT stage only. Additionally, the use of tools to cost the breeding program could potentially identify avenues to reallocate resources within a fixed budget for abiotic stress screening.

Within the IVT, there was a significant negative trend in plant height (-0.4 cm yr^-1^) and days to anthesis (-0.2 days yr^-1^) and a significant positive trend in moisture content (0.06% yr^-1^). The significant decrease in days to anthesis without a change in days to silking suggests anthesis-silking interval reduced over the study period. Increased flowering synchrony has been associated with maize yield gains in low yielding environments (Cairns et al., 2012; Das et al., 2018). Within the AVT, there was a significant positive trend in the number of ears per plant (0.01 yr^-1^), days to silking (0.1 days yr^-1^) and anthesis (0.1 days yr^-1^). While texture (-0.04 yr^-1^), stalk lodging (-0.02% yr^-1^), root lodging (-0.11% yr^-1^), severity of visual scores of MSV (-0.03 yr^-1^), GLS (-0.01 yr^-1^) and ear rots (-0.05 yr^-1^) showed negative trend. The higher number of traits that experienced significant changes over the past two decades in advanced varietal trials relative to intermediate trials is related to the higher number of traits measured in the AVT. Moisture content is, in part, a function of the time of harvest and significant changes in this trait are likely to be related, in part, to earlier harvesting of experiments.

The significant increase in tolerance to key diseases (except TLB) was relatively low using visual scores of disease severity. This could be attributed to both low heritability of disease scores and low disease pressure under natural infection. While artificially inoculated experiments accounted for 39%, 25% and 30% of experiments for MSV, TLB, GLS, respectively, artificial inoculation of diseases only started in 2012 and, except for TLB, was not used every year. Disease pressure was relatively low and artificial inoculation would help increase the pressure. The routine use of artificial inoculation for disease screening could be an important step towards increasing genetic progress for disease resistance. Since 2021 marker-assisted forward breeding for MSV resistance was implemented to enrich populations for MSV resistance prior to field phenotyping. Tolerance to both root and stalk lodging are essential traits used for product advancement in the DR&SS’s maize breeding pipeline. Estimated genetic trends showed a significant reduction in both root and stalk lodging during the study period, particularly root lodging, which reduced by almost two units during the study period. Plant height and ear height in the AVT were less than the mean of the IVT for both traits suggesting that there had been some selection for shorter stature. Interestingly, there was a significant trend towards a more flint endosperm (decrease in texture score). While in Malawi, the market demands flint maize varieties, with previous low market penetration of new improved maize varieties being linked to the release of dent varieties (Lunduka et al., 2012), there is no strong market preference in Zimbabwe where processing is through grain millers and not pounding by hand. The slight shift towards flint (-0.043 over the study period) was indirect, as selection pressure was not applied to grain texture. Interestingly, Tarekegne et al. (2023) showed a significant shift towards dent texture in the CIMMYT early southern Africa maize breeding pipeline. While currently, just under half (42%) of the hybrids sold in Zimbabwe are of flint texture, the ultra-early and early hybrid market is dominated by flint varieties, with 86% of hybrids in these market classes being flint.

Increasing genetic gain within the DR&SS maize breeding program is essential to deliver a steady stream of incrementally improved varieties to farmers as climate variability intensifies (Atlin et al., 2017). Compiling historical data provides an opportunity for breeding programs to review their current strategy. In this study, the proportion of candidate hybrids was just under 50% for approximately one-quarter of the years used in this study. Almost one-third of candidate hybrids within the AVT were tested for three or more years. Increasing the proportion of candidate hybrids relative to check varieties and reducing the number of years candidate hybrids are tested could allow the size of the breeding program to be expanded within the same budget. A further option to increase genetic gains under drought tolerance is by ensuring new trait donors with enhanced drought tolerance are incorporated in population improvement. Key heat and drought tolerant donors from the CIMMYT maize breeding program in India are currently being used in development of new populations to increase tolerance to drought and heat stress in southern Africa (Mukaro et al., 2023).

Increasing genetic gain is a function of both cost and time, yet most public crop breeding programs are faced with budgetary constraints (Cobb et al., 2019) and. It is therefore critical to consider reducing cost per unit gain as well as gain alone when assessing breeding program efficiency (Seck et al., 2023). Tools to fully cost breeding operations and pipelines, such as the University of Queensland Breeding Costing Tool (https://aussorgm.org.au/downloads/breeding-costing-tool/), combined with simulation tools (Faux et al., 2016) or deterministic models (Atlin and Econopouly, 2022) to compare alternative breeding strategies within a current pipeline budget should be considered.

## Conclusion

This is the first study to use historical data across two decades to document genetic trends in maize improvement within an African national program. Significant gains have been made within the DR&SS maize breeding pipeline over the past two decades; however, higher gains are required to meet projected food requirements in an increasingly variable climate, as the population expands. There is currently a strong push to modernize public breeding programs to increase breeding efficiency (Cobb et al., 2019; Covarrubias-Pazaran et al., 2021). Over the past few years the DR&SS maize breeding program has undergone significant changes including (i) defining the objectives of product profiles relative to the target market segments; (ii) modernization and adoption of new technologies that improve breeding efficiency; (iii) introduction of new favorable alleles through the selection of parents with high breeding value; (iv) application of doubled haploid technology to reduce the length of the breeding cycle, and (v) deployment of a breeding data management systems to reduce human error and support selection decisions. This study provides a benchmark to track the impact of these changes. Moving forward, quantifying genetic gain per unit cost rather than just genetic gain per se, is likely to be a more important indicator of breeding efficiency.

To date, the analysis of genetic trends using historical data have primarily been based on one testing stage of the breeding pipeline (Khanna et al., 2022; Prasanna et al., 2022; Rahman et al., 2023). However, the use of two stages (i.e., intermediate, and advanced variety trials) provided greater insight into gains at different stages of the breeding pipeline, highlighting where potential changes could be made to increase gains. Climate and crop modelling projections highlight that southern African maize-based systems are among the most vulnerable cropping systems to future climate change (Lobell et al., 2008; Tigchelaar et al., 2018). Therefore, it is essential that genetic trends with the DR&SS maize breeding pipeline not only continue but substantially increase.

## Data availability statement

The raw data supporting the conclusions of this article will be made available by the authors, without undue reservation.

## Author contributions

RM: Conceptualization, Data curation, Investigation, Methodology, Writing – original draft, Writing – review & editing. DC: Conceptualization, Data curation, Investigation, Methodology, Writing – original draft, Writing – review & editing. CS: Conceptualization, Data curation, Formal analysis, Investigation, Methodology, Software, Writing – original draft, Writing – review & editing. JC: Conceptualization, Investigation, Methodology, Writing – original draft, Writing – review & editing, Data curation. LM: Conceptualization, Data curation, Investigation, Methodology, Writing – original draft, Writing – review & editing. BP: Funding acquisition, Resources, Writing – review & editing. BM: Writing – review & editing. BD: Resources, Writing – review & editing, Funding acquisition. MM: Data curation, Writing – review & editing. WC: Writing – review & editing. XM: Data curation, Writing – review & editing. TN: Data curation, Writing – review & editing. NM: Data curation, Writing – review & editing. PM: Data curation, Writing – review & editing. DM: Data curation, Writing – review & editing. CMu: Data curation, Writing – review & editing. DW: Writing – review & editing. MZ-A: Writing – review & editing. CMa: Writing – review & editing. VC: Writing – review & editing. DK: Funding acquisition, Resources, Writing – review & editing, Project administration.
