# Supplementary material for: Genetic trends in the Zimbabwe’s national maize breeding program over two decades
Source: Front Plant Sci. 2024 Jun 5;15:1391926. doi: 10.3389/fpls.2024.1391926 (PMC11234322; doi:10.3389/fpls.2024.1391926)
Supplement: Supplementary file 1 [file Table_1.docx]

Supplementary Table 1. Key commercial hybrids used as checks in intermediate variety trials (IVT) and advanced variety trials (AVT) of the Department of Research & Specialist Services (DR&SS) maize breeding program. The first and last year of evaluation and their year of commercial release in Zimbabwe is also included.

| **Variety name** | **Company** | **Maturity** | **First year of evaluation** | **Last year of evaluation** | **Year of commercial release** |
| --- | --- | --- | --- | --- | --- |
| PAN413 | Pannar Seeds | Early | 2007 | 2021 | 1998 |
| PAN53 | Pannar Seeds | Early to Medium | 2006 | 2021 | 2007 |
| PAN5503 | Pannar Seeds | Early to medium | 2003 | 2012 | 2001 |
| PAN7M-97 | Pannar Seeds | Medium | 2007 | 2010 | 2007 |
| PGS61 | Klen Karoo | Medium | 2009 | 2018 | 2006 |
| PHB30G19 | Pioneer | Medium | 2008 | 2021 | 2008 |
| PHB30G97 | Pioneer | Medium | 2006 | 2019 | 2001 |
| SC513 | Seed Co | Early | 2003 | 2018 | 1999 |
| SC533 | Seed Co | Early | 2003 | 2014 | 2003 |
| SC627 | Seed Co | Medium | 2003 | 2019 | 1999 |
| SC633 | Seed Co | Medium | 2003 | 2014 | 2003 |
| SC635 | Seed Co | Medium | 2004 | 2014 | 2003 |
| SC637 | Seed Co | Medium | 2006 | 2019 | 2004 |
| ZAP51 | AgriSeeds | Early to medium | 2009 | 2014 | 2008 |
| ZAP61 | AgriSeeds | Medium | 2009 | 2019 | 2008 |
| ZS255 | Crop Breeding Institute | Medium | 2002 | 2015 | 1998 |
| ZS257 | Crop Breeding Institute | Medium | 2002 | 2011 | 1998 |
| ZS259 | Crop Breeding Institute | Medium | 2006 | 2014 | 2005 |
| ZS261 | Crop Breeding Institute | Medium | 2007 | 2018 | 2006 |
| ZS265 | Crop Breeding Institute | Medium | 2011 | 2021 | 2011 |
